# The Neuroanatomical Basis of Two Subcomponents of Rumination: A VBM Study

**DOI:** 10.3389/fnhum.2018.00324

**Published:** 2018-08-14

**Authors:** Emily L. L. Sin, R. Shao, Xiujuan Geng, Valda Cho, Tatia M. C. Lee

**Affiliations:** ^1^State Key Laboratory of Brain and Cognitive Sciences, The University of Hong Kong, Pokfulam, Hong Kong; ^2^Laboratory of Neuropsychology, The University of Hong Kong, Pokfulam, Hong Kong; ^3^Institute of Clinical Neuropsychology, The University of Hong Kong, Pokfulam, Hong Kong

**Keywords:** voxel-based morphometry, MRI, rumination, brooding, reflective pondering

## Abstract

Rumination is a trait that includes two subcomponents, namely brooding and reflective pondering, respectively construed as maladaptive and adaptive response styles to negative experiences. Existing evidence indicates that rumination in general is associated with structural and functional differences in the anterior cingulate cortex (ACC) and the dorsal lateral prefrontal cortex (DLPFC). However, conclusive evidence on the specific neural structural basis of each of the two subcomponents is lacking. In this voxel-based morphometry study, we investigated the independent and specific neural structural basis of brooding and reflective pondering in 30 healthy young adults, who belonged to high or low brooding or reflective pondering groups. Consistent with past research, modest but significant positive correlation was found between brooding and reflective pondering. When controlling for reflective pondering, high-brooding group showed increased gray matter volumes in the left DLPFC and ACC. Further analysis on extracted gray matter values showed that gray matter of the same DLPFC and ACC regions also showed significant negative effects of reflective pondering. Taken together, our findings indicate that the two subcomponents of rumination might share some common processes yet also have distinct neural basis. In view of the significant roles of the left DLPFC and ACC in attention and self-related emotional processing/regulation, our findings provide insight into how the potentially shared and distinct cognitive, affective and neural processes of brooding and reflective pondering can be extended to clinical populations to further elucidate the neurobehavioral relationships between rumination and prefrontal abnormality.

## Introduction

Rumination refers to a person indulging in passive and repetitive thinking on symptoms of distress and the possible causes and consequences of those symptoms (Nolen-Hoeksema and Morrow, 1993). Rumination is considered to be a trait-like construct and is present as a spectrum in the general population, with some individuals manifesting more prominent ruminative characteristics than others (Nolen-Hoeksema and Morrow, 1991; Just and Alloy, 1997; Spasojević and Alloy, 2001; Moberly and Watkins, 2008; Smith et al., 2009). Previous studies found that rumination is positively related to other personality traits such as neuroticism (Muris et al., 2005), and high ruminative level is associated with affective disorders such as depression and anxiety (Berman et al., 2011; Hamilton et al., 2011; Vanhalst et al., 2012). In essence, rumination characterizes individuals’ response and coping styles when faced with life distresses. According to the Response Style Theory, there are two subcomponents in rumination, namely brooding and reflective pondering, both of which can be quantitatively measured using the Response Style Scale (RSS).

Previous research has revealed a positive correlation between the two RSS subcomponents (Treynor et al., 2003; Nolen-Hoeksema et al., 2008). However, there are various differences between the two subcomponents in terms of their psychometric properties. First, brooding and reflective pondering were identified as two distinctive factors through factor analysis (Treynor et al., 2003). Moreover, brooding is positively and moderately associated with depression both concurrently and longitudinally, while reflective pondering is only associated with depression concurrently (Treynor et al., 2003). Conceptually, they may have some overlapping in initial processing of negative events but differ markedly in the characteristics of subsequent cognitive-affective processes. Specifically, brooding refers to the tendency to reflect on the (potential) negative impact of a current situation without devising a constructive solution (Treynor et al., 2003). Reflective pondering, on the other hand, is a tendency to contemplate the current situation with a constructive solution in mind and intentionally ponder one’s mind with a focus on problem solving (Treynor et al., 2003; Whitmer and Gotlib, 2013). In other words, during the brooding process, one is usually trapped in the affective loop and cannot move on to cognitive problem-solving, while during reflective pondering, one can successfully proceed to constructive cognitive processes directed at the problem at hand. Thus, brooding and reflective pondering were suggested to have different clinical implications, with brooding being considered a maladaptive form of coping strategy, while reflective pondering was suggested to be adaptive (Treynor et al., 2003).

A body of cross-sectional and longitudinal research has identified effects of personality traits such as neuroticism on gray matter volume (Blankstein et al., 2009; DeYoung et al., 2010; Taki et al., 2013). These studies show that it is valuable to understand the neuroanatomical basis of individual differences in personally traits. However, existing research on the neural basis of rumination trait is limited. One recent study found that gray matter volume of the dorsolateral prefrontal cortex (DLPFC) positively predicted rumination in non-depressed individuals, which was interpreted as potentially reflecting functional inefficiency and overloading (Wang et al., 2015). In another study, self-reported rumination was positively predicted by resting-state functional connectivity between the DLPFC and the rostral anterior cingulate cortex (ACC) in depressed patients, which in turn was positively correlated with the DLPFC cortical thickness (Späti et al., 2015). Collectively, existing evidence suggests that the DLPFC and ACC gray matter structures are most commonly associated with self-reported rumination (Pizzagalli, 2011; Ghaznavi and Deckersbach, 2012; Kühn et al., 2012; Wang et al., 2015). Indeed, the total score of rumination showed negative correlation with left ACC gray matter volume among healthy participants even after controlling for depressive symptomology (Kühn et al., 2012), underscoring the integral role of the ACC in ruminative processes (Pizzagalli, 2011). However, brooding and reflective pondering were not separately investigated in Kühn et al. (2012), hence the relationship between ACC or DLPFC gray matter volume and each of the two subcomponents of rumination remains unclear.

The DLPFC was proposed to perform memory and attention functions, as well as cognitive manipulation of incoming information (Dixon et al., 2017). Consistent with this, depressive patients show both DLPFC structural abnormalities and functional hypoactivity in resting-state and task-based fMRI (Gotlib and Hamilton, 2008; Koenigs and Grafman, 2009). Among healthy participants, in task-based fMRI, healthy subjects with high brooding tendency showed more DLPFC activations when trying to disengage from negative information compared to those with low brooding tendency (Vanderhasselt et al., 2011, 2013). Similarly, among major depressive patients, rumination induction elicited greater activations in the DLPFC than a distraction task (Cooney et al., 2010). The ACC is important for encoding affective value as well as in emotion regulation and cognitive control (Mohanty et al., 2007). Greater activations in the rostral ACC (rACC) were found while participants were performing an emotional rather than cognitive stroop task (Mohanty et al., 2007). Further, when performing an emotion contrast task, dorsal ACC activity predicted individual differences in brooding score (Vanderhasselt et al., 2013).

Our study specifically investigated the independent neuroanatomical (gray matter volume) basis of the two rumination subcomponents to elucidate the underlying common and distinct cognitive and affective mechanisms of brooding and reflective pondering. Based on existing literature on important roles of the DLPFC and the ACC on cognitive executive control and affect processing/regulation, we focused on those two areas as regions of interest (ROI). We hypothesized: (1) a positive relationship between scores on brooding and reflective pondering; (2) gray matter volumes in the DLPFC and ACC would be larger for high than low brooders. Given previous evidence suggesting opposite clinical implications for brooding (maladaptive) and reflective pondering (adaptive), we tentatively hypothesized that; and (3) reflective pondering would show opposite (i.e., negative) relations with DLPFC and ACC gray matter volume to brooding.

## Materials and Methods

### Participants, Measures and Procedure

Thirty healthy, right-handed, Chinese adults (11 males and 19 females) aged between 20 years and 48 years old (Mean = 31.77 years; SD = 6.84 years) participated in this study. These participants were recruited from the community. Participants did not have any current or prior history of neurological or psychiatric disorders that might affect their cognitive functioning. The current study was approved by the Institutional Review Board of The University of Hong Kong. All participants signed informed consents prior to participation.

The Ruminative Response Scale (RRS; Nolen-Hoeksema and Morrow, 1991) is a 22-item self-rating scale designed to assess an individual’s propensity to ruminate. It consists of two subscales that purport to measure the two subcomponents of rumination, namely brooding and reflective pondering. The items are mainly self-relevant and focus on the possible antecedents and consequences of one’s depressed mood state, which can be used to ascertain how an individual generally feels, thinks, and reacts when feeling down or depressed.

The Chinese version of the 14-item Hospital Anxiety and Depression Scale (HADS; Leung et al., 1999) was used to measure the severity of depression (HADS-D) and anxiety (HADS-A) in the participants. The HADS is a widely used scale in measuring the severity of depression and anxiety symptoms in both psychological and neuropsychiatric studies. There are two subscales, anxiety and depression, containing seven questions in each. Scores range from 0 to 21 and are categorized as follows: normal 0–7, mild 8–10, moderate 11–14 and severe 15–21 (Whelan-Goodinson et al., 2009). The Chinese version of HADS has good internal consistency (Cronbach alpha = 0.86; Leung et al., 1999).

Through employing a median split procedure on the Brooding and Reflective subscales of the RRS, all participants were assigned to either High Brooding Group (HBG) or Low Brooding Group (LBG) and either High Reflective Pondering Group (HRG) or Low Reflective Pondering Group (LRG). The Brooding and Reflective scores showed modest but significant correlation (*r* = 0.394; *p* = 0.031).

In total, 14 participants (five males; mean age = 30.57 years) were assigned to the LBG (brooding score ≤9; Mean = 7.86; SD = 1.02), whereas the remaining 16 participants were assigned to the HBG (six males; mean age = 32.81 years; brooding score >9; Mean = 12.81; SD = 2.43). The high and LBGs only differed in terms of the brooding levels (*t* = −7.44, *p* < 0.001), but not in age (*t* = −0.90, *p* = 0.644), gender composition (*X*^2^ = 0.010, *p* = 0.919), self-reported level of depression or anxiety (|*t*| < 1.17, *p*s > 0.25), or reflective pondering score (*t* = −1.820, *p* = 0.080).

In a similar vein, the same 30 participants were divided into LRG (reflective pondering score ≤9; 15 subjects with three males; mean age = 30.87 years) and HRG (reflective pondering score >9; 15 subjects with eight males; mean age = 32.67 years) based on their scores on the Reflective Pondering subscale of the RRS. Again, no significant between-group difference was identified in demographic characteristics (age: *t* = −0.714, *p* = 0.481; gender composition: *X*^2^ = 3.589, *p* = 0.128), self-reported level of depression or anxiety (|*t*| < 0.44, *p*s > 0.66). Also, the HRG scored significantly higher than the LRG group only on the Reflective Pondering subscale of the RRS (*t* = −8.735, *p* < 0.001), but not on the Brooding subscale (*t* = −1.614, *p* = 0.118).

On the study day, each participant was instructed to complete the self-reported questionnaires including the RRS and HADS, before entering the MRI scanner.

### Image Acquisition

High-resolution anatomical images were acquired via a 3.0 Tesla Philips Medical Systems Achieva scanner with an eight-channel SENSE head coil. A three-dimensional, T1-weighted, magnetization-prepared rapid acquisition gradient-echo sequence was used with 164 contiguous sagittal slices (time to repetition = 7 ms; time to echo = 3.2 ms; acquisition matrix = 240 × 230; sagittal field of view = 164 mm; flip angle = 8°; voxel size = 1 × 1 × 1 mm^3^).

### Structural Brain Image Pre-processing and Analysis

The CAT12 toolbox^1^ within SPM12 (FIL, London, UK) in MATLAB 7.12.0 environment (Mathworks Inc., Natick, MA, USA) was used to preprocess the MRI images. The T1 images were manually reoriented and centered on the anterior commissure as the point of origin. Each T1 image was then visually inspected in SPM12 to check for any artifacts or gross anatomical abnormalities. Next, the T1 images were segmented into six tissue types and normalized to the standard MNI template through the DARTEL procedure using customized template. Segmentation and normalization quality was manually inspected for all participants. Finally, the resulted modulated normalized gray matter images were smoothed with a standard Gaussian kernel of 8-mm FWHM.

The smoothed gray matter images were then used for subsequent imaging analyses in SPM12. Two general linear models (independent-samples *t*-tests) were used to examine the respective effect of brooding group (LBG vs. HBG) and reflective pondering group (LRG vs. HRG), while controlling for the other component (reflective pondering score in the former case and brooding score in the latter case). As presented above, the brooding and reflective pondering continuous scores significantly correlated with each other (*r* = 0.394; *p* < 0.05), meaning that the analysis power for detecting the effect of either variable would be markedly reduced if both were included simultaneously in the model. However, the correlations between brooding group and reflective pondering score, and between reflective pondering group and brooding score, were not significant (*p*s ≥ 0.08). Thus, for the whole-brain imaging analysis with relatively stringent statistical correction thresholds and limited power, we decided to use the dichotomous brooding (and reflective pondering) group variable when investigating their effects, while keeping them as continuous when they entered the model as nuisance variables, in order to achieve a balance of analysis power and complete control of nuisance effects. For completeness, a multiple regression analysis was also performed on the T1 images in which both the brooding and reflective pondering scores were entered as continuous variables, and results of this analysis were compared to those of the group-based analyses outlined above. Age, gender and total intracranial volume (TIV) were also entered as additional nuisance variables. We primarily focused on two *a priori* ROIs, namely the DLPFC and ACC, which were constructed using WFU_Pickatlas software based on Talairach Daemon atlas. Both masks were bilateral. Within those ROIs, small-volume correction tests were conducted. Through conducting the ROI-based analyses, we ensured that any DLPFC or ACC clusters that we observed were anatomically confined to the respective structure. Complementary whole-brain analyses were also performed. To account for Type-I errors, the results threshold were set at uncorrected *p* < 0.001 at voxel level, and FWE-corrected *p* < 0.05 at cluster level, within the searching space of either the ROIs or across the whole brain.

### Statistical Analyses

Correlation analysis was conducted in SPSS 20.0 (IBM, Armonk, NY, USA) to examine the relationship between brooding and reflective pondering scores. To further characterize the independent associations between the two subcomponents of RRS and gray matter volume, we extracted the average gray matter values from the significant clusters resulted from the independent-samples *t*-tests, and conducted a set of univariate ANOVA assessing the effects of brooding and/or reflective pondering groups while controlling for age, gender, TIV and the alternative RRS subscale score. For completeness, we also replicated the significant results with both brooding and reflective pondering scores as continuous independent variables in multiple-regression analyses. Notably, the ANOVA analyses are not independent from the whole-brain analyses, but it rather served the function of checking whether the regional gray matter volume that was significantly affected by brooding (or reflective pondering) at the whole-brain level would also show some levels of relation with the alternative subscale. All statistical results were evaluated at *p* < 0.05, two-tailed.

## Results

### Psychometric Analysis

**Figure 1:**
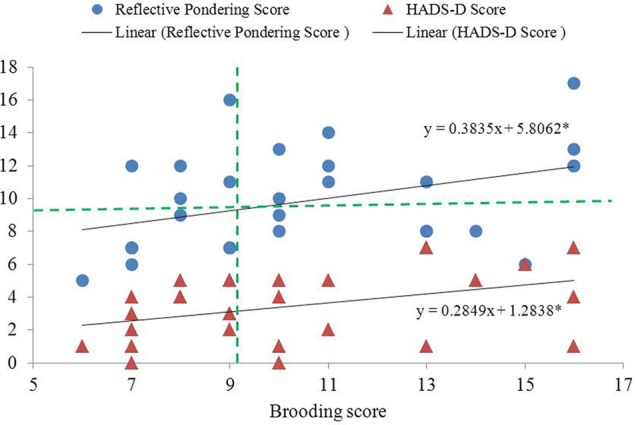
Significant inter-correlation between the brooding and reflective pondering subscales of the rumination response scale (RRS) questionnaire, and between brooding and HADS-D. Brooding score showed significant and positive correlation with both reflective pondering and HADS-D scores (controlling for reflective pondering score). The two dashed lines indicate cut-off points for dividing low- and high-brooding (vertical line) and reflective pondering (horizontal line) groups. *Significant at *p* < 0.05, 2-tailed.

The two subcomponents of the RRS were significantly and positively correlated (*r* = 0.394, *p* = 0.031; Figure 1). Participants’ HADS-D (mean = 3.46, SD = 2.12) and HADS-A scores (mean = 4.21, SD = 2.69) were significantly and marginally correlated with their brooding scores (*r* = 0.402 and 0.342, *p* = 0.038 and 0.081, respectively) after controlling for reflective pondering scores (Figure 1). In contrast, neither HADS-D nor HADS-A scores correlated with reflective pondering scores after controlling for brooding scores (|*r*| < 0.11, *p*s > 0.58).

### Imaging Analysis

**Figure 2:**
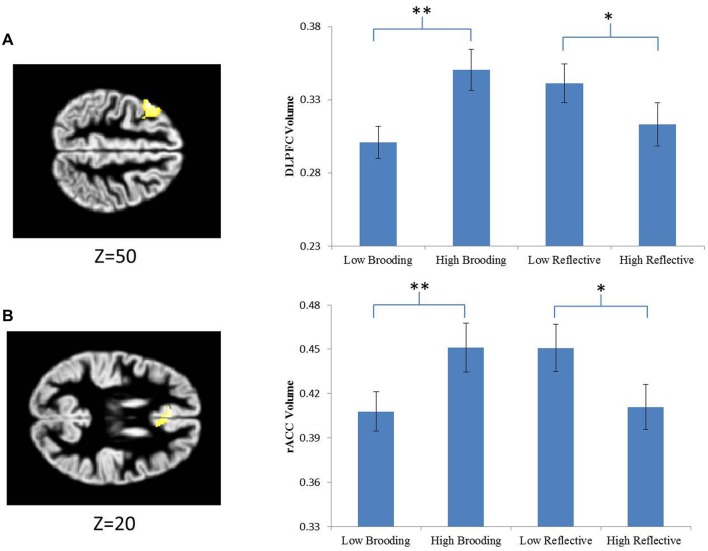
Extracted parameter estimates of significant clusters to the effect of brooding. Signals were extracted from two *a priori* regions of interests (ROIs). **(A)** The dorsal lateral prefrontal cortex (DLPFC) and **(B)** the anterior cingulate cortex (ACC). The significant clusters (DLPFC and rostral ACC) are overlaid on standard anatomical templates. MNI z coordinates are provided below the axial slices. *Indicates statistically significant effects at *p* < 0.05, **indicates statistically significant effects at *p* < 0.001.

ROI analysis revealed a significant main effect of brooding group on both DLPFC and ACC gray matter volumes, with the HBG showing increased gray matter than the LBG (DLPFC: peak coordinate = −48, 15, 50, max *t* = 5.73, cluster size = 762 voxels, FWE-corrected *p* = 0.005; ACC: peak coordinate = −9, 39, 20, max *t* = 4.69, cluster size = 915 voxels, FWE-corrected *p* < 0.001; Figure 2). The ACC cluster remained significant in the multiple regression analyses in which both brooding and reflective pondering scores were entered as continuous variables (max *t* = 4.54, cluster size = 247, FWE-corrected *p* = 0.017), while the DLPFC cluster no longer survived cluster-based FWE correction. However, the DLPFC cluster survived peak-level FWE correction in the multiple regression analysis (max *t* = 5.11, FWE-corrected *p* = 0.031, cluster size = 53). Whole-brain analysis revealed no significant cluster. We also found no cluster that survived correction at whole-brain or ROI-level for the effect of reflective pondering group.

The mean gray matter values of the significant DLPFC and ACC clusters were extracted and subjected to further complementary ANOVA analyses in SPSS. As expected, significant effects of brooding group were observed for both clusters (DLPFC: *F*_(1,24)_ = 30.68, *p* < 0.001; ACC: *F*_(1,24)_ = 23.538, *p* < 0.001). However, reflective pondering also showed significant, albeit quantitatively smaller, effects on both clusters (DLPFC: *F*_(1,24)_ = 5.671, *p* = 0.026; ACC: *F*_(1,24)_ = 8.972, *p* = 0.006). Thus, the two rumination subcomponents showed opposite relations with the gray matter volumes of the same ACC and DLPFC regions, with the effect of reflective pondering being smaller in magnitude and undetected at the whole brain/ROI level. Quantitatively similar results were obtained if both brooding and reflective pondering scores were entered as continuous variables in a multiple-regression analysis (for brooding: *t*s ≥ 2.64, *p*s ≤ 0.014; for reflective pondering: *t*s ≤ −2.34, *p*s ≤ 0.028). No significant interactive effect of the two RSS subscale scores was observed on either DLPFC or ACC gray matter volume (*p* > 0.9). Further *post hoc* analyses revealed that while the HBG showed increased gray matter in both DLPFC and ACC than the LBG (DLPFC: *t*_(25)_ = 5.405, *p* < 0.001; ACC: *t*_(25)_ = 4.852, *p* < 0.001), the HRG showed gray matter reductions in both DLPFC and ACC than the LRG (DLPFC: *t*_(25)_ = −2.332, *p* = 0.029; ACC: *t*_(25)_ = −2.923, *p* = 0.008; Figure 2).

## Discussion

In this study, we confirmed that in healthy individuals, brooding and reflective pondering showed modest positive correlation with each other. However, while brooding showed independent positive effect on gray matter volumes of the left DLPFC and rostral ACC, reflective pondering showed independent negative effect on those structures. Thus, our results suggest that while the two subcomponents of rumination might share some common processes, they appear to have distinct and potentially even opposite underlying neural mechanisms.

### Conceptual Overlapping of Brooding and Reflective Pondering

We found a positive correlation between the scores of the two subcomponents of RSS. Consistent with previous studies, positive relationship between the two subcomponents may reflect the conceptual overlapping between them (Nolen-Hoeksema and Morrow, 1993; Treynor et al., 2003). As explained in the introduction, brooding refers to the process that one focuses on the cause and consequence of his/her negative experiences without engaging in constructive problem-solving, while reflective pondering is a tendency to contemplate the current situation with a constructive solution in mind. Conceptually, both types of ruminative processes start with attending closely to the negative events. Thus, the positive correlation found likely reflects the common initial attentive processes to negative stimuli (Nolen-Hoeksema and Morrow, 1991, 1993). However, brooding and reflective pondering styles diverge in subsequent cognitive and emotional processes, as discussed below.

### Brooding, DLPFC and ACC

Our findings indicate that participants with higher brooding tendency also showed larger left DLPFC gray matter volume. The DLPFC is strongly implicated in working memory and attention processes, as well as in manipulation of incoming information (Miller and Cohen, 2001; Dixon et al., 2017). Reduction of DLPFC volume has been found in patients with major depressive disorder (Grieve et al., 2013; Lai, 2013). More specifically, the left DLPFC was proposed to be specifically involved in responding to positively-valenced stimuli (Pizzagalli et al., 2005; Balconi and Ferrari, 2012a,b), and reduced left vs. right lateral PFC activation may underlie the affect regulation deficits in depressed individuals (Mathersul et al., 2008; Briceño et al., 2013). Given brooding refers to a tendency to maintain attention to negative stimuli (Joormann et al., 2006), high-brooders would tend to experience greater difficulties in disengaging from negative information, possibly due to lower efficiency of DLPFC functioning. Thus, among high-brooders, the left DLPFC may be recruited to greater extents to compensate for this inefficiency (Vanderhasselt et al., 2011, 2013; Wang et al., 2015), possibly through upregulating positive affective processing, which then gradually lead to increase in DLPFC structural volume (Draganski et al., 2004; Scholz et al., 2009; Wang et al., 2015). Failure to engage in this compensatory process could lead to greater affective dysregulation and more negative affective states, coupled with reduced DLPFC volume as observed in major depressive patients (Grieve et al., 2013; Lai, 2013). In our study, brooding and affective symptomology were positively correlated, suggesting that high brooding tendency constitutes core characteristics of affective dysregulation. Furthermore, the association between brooding and depressive scores appeared quantitatively stronger than that between brooding and anxiety scores, consistent with existing research that while rumination may be specifically associated with depression, anxiety may exhibit a stronger relationship with worry (Hong, 2007; Yook et al., 2010). Such associations need to be further tested in clinical/subclinical samples. Since our high-brooding participants were still free of clinical affective disorders, increased left DLPFC volume might be a key adaptive mechanism that protected those individuals from developing clinically significant affective conditions. It is worth noting that subtle difference in the brooding effect on DLPFC volume was observed depending on whether brooding score was dichotomized or entered as continuous variable, such that the effect survived the predefined cluster-level correction in the former case but only survived peak-level correction in the latter case. The lack of statistical power due to modest inter-correlation between the brooding and reflective pondering scores may partly contribute to this discrepancy. Alternatively, there could be inter-participant heterogeneities within the high-brooding group in engagement of compensatory processes for the dysregulated cognitive-affective system. Moreover, it may be that the relationship between brooding score and DLPFC gray matter is not entirely linear across the full spectrum of brooding tendency. These possibilities need to be tested in future research involving larger participant samples.

We also found a positive association between high brooding tendency and ACC gray matter volume, regardless of whether brooding score was dichotomized or assessed as a continuous variable. The rostral ACC is considered to be responsible for assessment of emotional information and regulation of emotional responses (Mohanty et al., 2007). As part of the default mode network, the rostral ACC is also heavily involved in self-referential processing (Nejad et al., 2013), which is an integral component of rumination. Specifically, brooding was suggested to be similar to analytic self-focusing processes that are associated with clinical affective symptoms and poor problem-solving skills (Nejad et al., 2013). Thus, the increased ACC volume in high-brooders may be a long-term consequence of heightened engagement in self-referential affective processing (Pizzagalli, 2011). Moreover, brooding is associated with negative attention biases, and the increased in ACC volume may reflect a shift towards general hypervigilance and reactivity to negative affective stimuli (Boes et al., 2008). On the other hand, gray matter volume of the ACC was found to be significantly reduced in both major depression and bipolar disorder (Drevets et al., 1997, 2008; Grieve et al., 2013; Lai, 2013). In this regard, the increased ACC structural volume, which could be a long-term consequence of greater recruitment of this region in performing affect regulatory functions (Draganski et al., 2004; Scholz et al., 2009), could be an adaptive mechanism that protected the high-brooding participants from developing clinical affective disorders.

### Reflective Pondering, DLPFC and ACC

Although we did not find significant gray matter volume differences between high- and low-reflective pondering groups at the whole-brain level, we observed negative effects of reflective pondering on both left DLPFC and ACC gray matter volumes which showed positive effects of brooding. These results indicate that reflective pondering has distinct and opposite neural basis to brooding. As discussed above, during the initial stage of information processing, both high-brooders and high-reflective ponderers may show heightened attention towards negative stimuli (Vanderhasselt et al., 2013; Whitmer and Gotlib, 2013). However, during subsequent cognitive and affective processing, high-reflective pondering individuals may show better ability in disengaging from negative self-focused affective processing towards problem-focused cognitive processing. In this regard, high-reflective ponderers can be considered as manifesting relatively high levels of early-stage negative affective bias *as well as* high later-stage negative affect regulatory capacity, which might explain why the DLPFC and ACC volumetric difference between the high- and low-reflective pondering groups was not as prominent as that between high- and low-brooding groups. Consistent with this reasoning, existing evidence indicates that reflective pondering is unrelated to cognitive biases after controlling for brooding and depressive scores in healthy subjects, suggesting that unlike the maladaptive style of brooding, reflective pondering encompasses certain adaptive mechanisms that reduce the prolonging affective impact of negative stimuli (Joormann et al., 2006). In this vein, individuals scoring higher on reflective pondering might be considered to have greater efficiencies in cognitive and affect regulatory functions performed by the DLPFC, which would explain the relatively lower DLPFC volumes in those individuals. Such speculation needs to be formally tested by future functional network connectivity studies to clarify the intricate relationships between DLPFC structural volume and functional connectivity/efficiency, rumination and affect regulatory functioning.

Likewise, reflective pondering showed a negative relationship with rostral ACC volume. The rostral ACC is important in regulating self-referential affective processes. As individuals scoring higher in reflective pondering tend to more quickly disengage from negative self-focused processing, there is less need for those individuals to recruit the rostral ACC, leading to the long-term consequence of relatively lower ACC structural volume. A related possibility is that high-reflective ponderers are more efficient in affect regulatory functions performed by the rostral ACC; therefore, the structural volumes of the ACC are relatively small in those individuals. These speculations again need to be formally tested in future functional activation and connectivity studies.

## Limitations

Our study has several limitations. First, the current study adopted a cross-sectional design, which precluded us from determining the directionality of the brain-emotion relationship. It remains unknown whether the changes in brain structures preceded or followed the development of rumination response style. Second, in view of our modest sample sizes, the current results should be interpreted with caution and replicated on larger samples. Third, we did not include complementary questionnaires (other than the HADS) to establish the external construct validity of the rumination subscales. Future study could include other trait scales like the Neuroticism scale from NEO Personality Inventory. Last but not the least, future studies may include patients with affective disorders to further advance our understanding on the emotional, cognitive and neural mechanisms of brooding and reflective pondering during the course of clinical conditions. These insights are critical to the development of comprehensive intervention programs.

## Conclusion

This study provides important evidence that among healthy adults, the two subcomponents of rumination, namely brooding and reflective pondering, respectively showed independent positive and negative effect on gray matter volumes of the left DLPFC and ACC, areas that have been implicated in attention, affect reactivity/regulation, and self-referential processes. Based on these results, we propose that while brooding and reflective pondering might share some initial attentional processes to negative stimuli, these two different rumination styles show distinct subsequent cognitive and affect regulatory processes, as reflected by their distinct neural structural basis, which in turn have different clinical significances. These findings provide new and important insights into the neurocognitive processes of the rumination sub-processes, which can be extended to clinical populations to further elucidate the neurobehavioral and potential adaptive effects of rumination styles and prefrontal abnormality.

## Author Contributions

VC and TL conceptualized the study. VC collected the data. ES, RS and XG analyzed the data. ES, RS, XG and TL wrote the manuscript. All authors read and approved the final version of the manuscript and agreed with its submission to Frontiers in Human Neuroscience.

## Conflict of Interest Statement

The authors declare that the research was conducted in the absence of any commercial or financial relationships that could be construed as a potential conflict of interest.

## Footnotes

^1^http://dbm.neuro.uni-jena.de/vbm
